# Intratumoral treatment with radioactive beta-emitting microparticles: a systematic review

**DOI:** 10.1007/s13566-017-0315-6

**Published:** 2017-06-24

**Authors:** Robbert C. Bakker, Marnix G.E.H. Lam, Sebastiaan A. van Nimwegen, Antoine J.W.P. Rosenberg, Robert J.J. van Es, J. Frank W. Nijsen

**Affiliations:** 1 Department of Radiology and Nuclear Medicine, University Medical Center Utrecht, Heidelberglaan 100, 3584 CX Utrecht, The Netherlands; 2 Department of Oral and Maxillofacial Surgery, University Medical Center Utrecht, Utrecht, The Netherlands; 3 Department of Clinical Sciences of Companion Animals, Faculty of Veterinary Medicine, Utrecht University, Utrecht, The Netherlands; 4 Department of Head and Neck Surgical Oncology, UMC Utrecht Cancer Center, Utrecht, The Netherlands

**Keywords:** Microbrachytherapy, Injection, Microspheres, Particles, Brachytherapy, Selective internal radiation therapy

## Abstract

**Purpose:**

The purpose of this study was to review the role of radioactive microparticles (1–100 μm) for the treatment of solid tumors and provide a comprehensive overview of the feasibility, safety, and efficacy.

**Methods:**

A systematic search was performed in MEDLINE, EMBASE, and The Cochrane Library (January 2017) by combining synonyms for the determinants “tumor,” “injection,” and “radionuclide.” Data on injection technique, toxicity, tumor response, and survival were collected.

**Results:**

The search yielded 7271 studies, and 37 were included for analysis. Twelve studies were performed in human patients and 25 animal studies. The studies were heterogeneous in patient population, tumors, follow-up time, and treatment characteristics. The direct intratumoral injection of radioactive microparticles resulted in a response rate of 71% in a variety of tumors and uncomplicated procedures with high cumulative doses of >19,000 Gy were reported.

**Conclusion:**

The large variety of particles, techniques, and treated tumors in the studies provided an important insight into issues concerning efficacy, safety, particle and isotope choice, and other concepts for future research. Animal studies showed efficacy and a dose response. Most studies in humans concluded that intratumoral treatment with radioactive beta-emitting microparticles is relatively safe and effective. Conflicting evidence about safety and efficacy might be explained by the considerable variation in the treatment characteristics. Larger particles had a better retention which resulted in higher anti-tumor effect. Leakage seems to follow the path of least resistance depending on anatomical structures. Subsequently, a grid-like injection procedure with small volume depots is advised over a single large infusion. Controlled image-guided treatment is necessary because inadequate local delivery and inhomogeneous dose distribution result in reduced treatment efficacy and in potential complications.

## Introduction

Interventional oncology is an emerging field in cancer care that has the potential to complement existing treatment modalities. Today, various image-guided interventions have an active role in the palliative cancer treatment setting [1–3]. Driven by technical innovation, new image-guided treatment solutions are continuously developing. Interventional oncology techniques, using microspheres or “microbrachytherapy,” have potential benefits, including minimal invasive delivery, outpatient treatment, and improved (progression-free) survival and quality of life [4, 5]. The high-absorbed dose of beta-radiation enables a local tumor-ablative effect while the limited penetration depth of maximum 2–11 mm (Table 1) minimizes side effects.

**Table 1:** Characteristics of radionuclides in microparticles

Radionuclide		Half-life (days)	Beta energy (MeV)		Tissue penetration (mm)		Gamma energy		Production method
			Mean	Maximum	Mean	Maximum	keV	% Decay	
Phosphorus-32	^32^P	14	0.695	1710.6	2.9	8	–	–	Reactor
Yttrium-90	^90^Y	2.7	0.935	2280.1	3.9	11	–	–	Reactor or strontium-90/yttrium-90 generator
Iodine-131	^131^I	8.0	0.182	806.9	0.9	5	365	82%	Reactor
Holmium-166	^166^Ho	1.1	0.666	1854.9	3.2	9	81	6.7%	Reactor
Rhenium-186	^186^Re	3.8	0.362	1069.5	1.8	7	137	9.8%	Reactor
Rhenium-188	^188^Re	0.71	0.764	2120.4	3.5	10	155	15.6%	Tungsten-188/rhenium-188 generator

*MeV* mega electron volt

The aim of this literature study was to review the potential role of beta-emitting microparticles for intratumoral (IT) treatment of solid malignant neoplasms. A comprehensive overview of the technical aspects and the characteristics of commonly used radionuclides are provided. Finally, recommendations for further investigation are formulated.

## Methods

### Protocol and registration

Methods of the analysis and inclusion criteria were specified in advance and documented in a protocol registered in an international prospective register of systematic reviews (PROSPERO) [6].

### Eligibility criteria

**Fig. 1:**
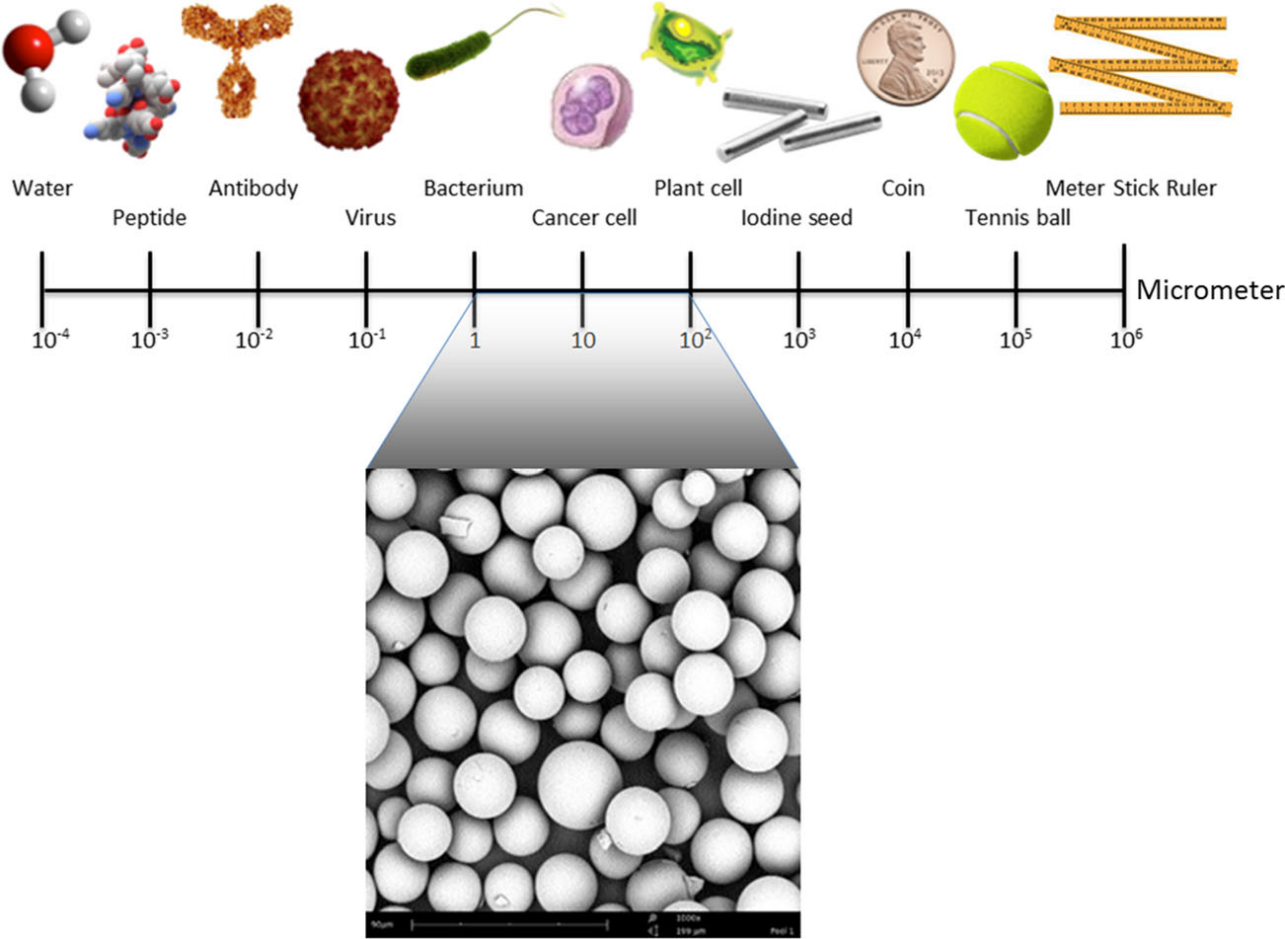
Illustration of particle size. Particles sized between 1 and 100 μm fulfilled our definition of microparticles, as compared to smaller carriers like antibodies for radioimmunotherapy or nanoparticles, and larger particles or seeds for conventional brachytherapy

Type of studies: There were no restrictions based on study design, setting, timing, and publication date or publication status. Only full-text articles reported in the English language were included. Studies that examined human or veterinary patients or animal models with solid tumors were included. There were no restrictions on tumor size, type, or location. The administration of the radioactive microparticles had to be performed directly into the tumor. Particles sized between 1 and 100 μm fulfilled our definition of microparticles (Fig. 1), and the particles had to emit beta-radiation. Combined treatment regimens with external beam radiotherapy (EBRT) or chemotherapy were also included. Local treatments after incomplete tumor resections were excluded.

Endpoints included technical details (particle size, injection method, and the amount of injection fluid), biodistribution (retention, IT distribution, and leakage of activity), safety (local and systemic adverse events), and efficacy.

### Search

For this review, the electronic databases MEDLINE, EMBASE, and Cochrane Library were searched from dates of inception until January 1, 2017. To ensure literature saturation, the reference lists and citing articles of included studies or relevant reviews identified through the search were scanned.

The full search strategy is listed in the Appendix.

### Study selection

The records derived from the search were assessed for eligibility by the author (R.B.) on the titles and abstracts. Full-text manuscripts were screened for all titles that met the inclusion criteria. The reasons for exclusion were recorded. The risk of bias was assessed according to the Newcastle-Ottawa scale to ascertain the validity of eligible trials [7].

### Data extraction

The data included (1) methodology, (2) participant details, (3) intervention details, and (4) treatment effect and side effects.

## Results

**Fig. 2:**
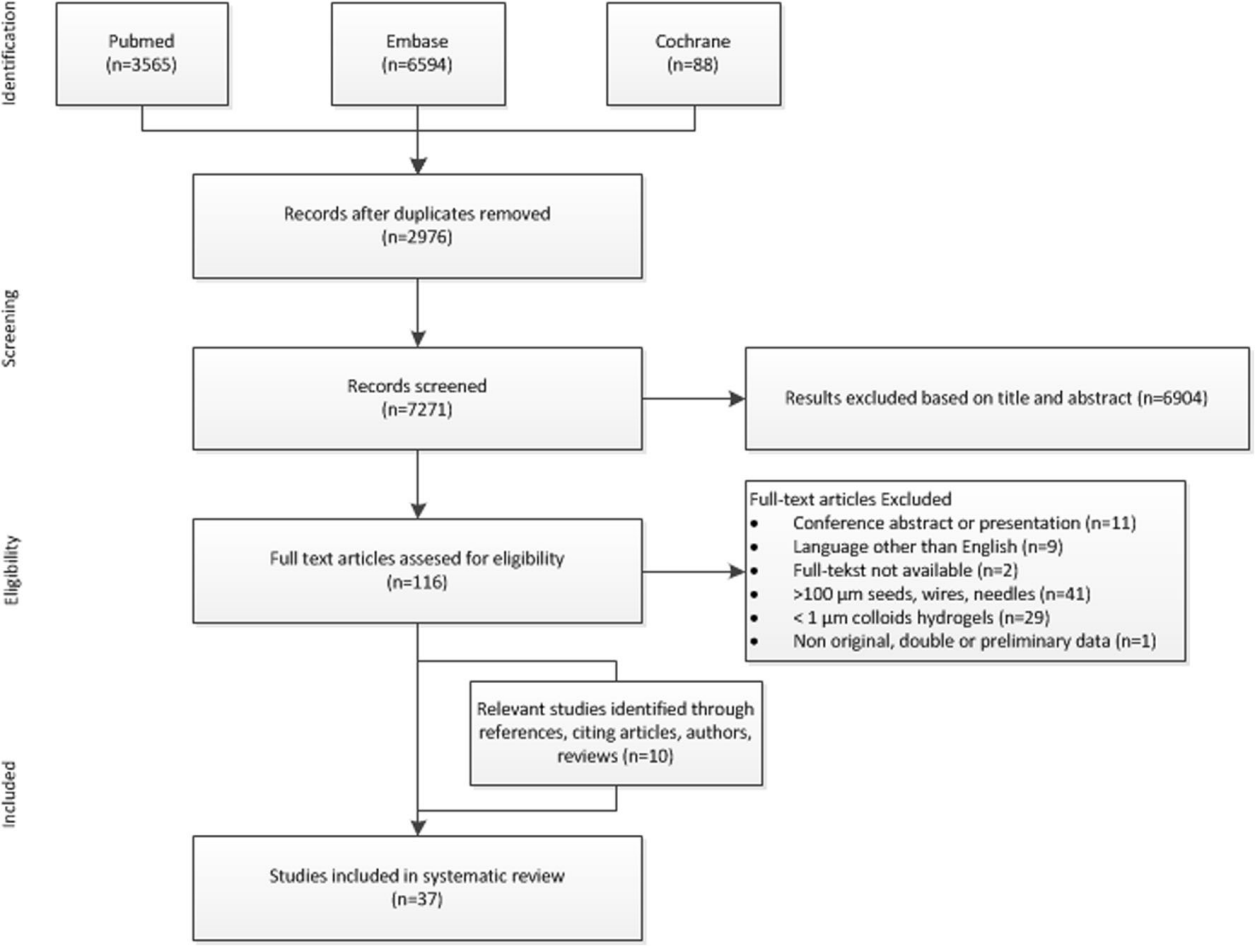
Flow diagram of article selection

After the removal of duplicates, 7271 records remained out of 10,247 initial records. Of these, 7151 publications did not meet the criteria after reviewing the title and abstract. Subsequently, 22 of the 120 publications were discarded because full text was not available (*n* = 2), not in the English language (*n* = 9), or conference abstract or poster (*n* = 11). The full texts of the remaining 98 studies revealed another 68 studies that did not meet the inclusion criteria. Two additional studies were excluded because of preliminary data and double publication. Cross-referencing identified nine additional studies that fulfilled the inclusion criteria. A total of 37 studies (performed between 1962 and 2014) were included in this review (Fig. 2).

### Characteristics and quality of included studies

Twelve studies described the use of beta-emitting microparticles in humans, 23 studies a single animal model, a single study two species, and a single study was performed in veterinary patients. In humans, only one randomized clinical trial was conducted, six cohort studies and five case series. In total, 183 human patients were treated, including a large variety of malignancies, all refractory to other treatments. The used animals in the tumor model studies were mice (13/24), rats (10/24), and rabbits (2/24). Microbrachytherapy in animals was performed in relatively small tumors (±1 cm), as larger tumors are considered not ethically feasible in small rodent models. The tumors were implanted subcutaneously (*n* = 15) or in the organ of origin (orthotopically) (*n* = 7), or were chemically induced (*n* = 2) [8, 9]. Furthermore, a case series of three feline veterinary patients with a large spontaneous tumor in the liver were treated [5]. The quality of evidence was poor, primarily by design and number of participants. Furthermore, the large variety of microparticles, treatment methods, tumor type, and location made a proper systematic comparison impossible. Therefore, a more descriptive approach was necessary.

### Type of microparticles

Microspheres (MS) [10–14] and chromic phosphate particles (CPP) were used in five and seven human studies, respectively [15–21] (Table 2). In the 25 animal studies including the study in veterinary patients, a similar division between MS [5, 22–35] (*n* = 15) and CPP [8, 9, 36–40] (*n* = 7) was made, with some additional microparticles like ^188^Re sulfide particles (*n* = 2) [41, 42] and labeled MAA particles [43] (Table 3).

**Table 2:** Technical details of human studies

Study	Number of patients	Tumor type	Isotope	Particle	Particle size (μm)	Multiple Injections/single infusion	Imaging	Needle gauge	Tumor size^a^	Amount of fluid	Injected activity (MBq)/absorbed dose (Gy)
Kim 1962 [10]	10	Breast, bladder, brain, prostate, lung, metastasis	^90^Y	Ceramic MS	60 ± 5	Multiple injections					
Blanchard 1965 [11]	12	Bladder, prostate, breast, lung, metastasis	^90^Y	Ceramic MS	40–60	Multiple injections					925–11,100 MBq
Ariel 1978 [12]	1	Rhabdomyosarcoma	^90^Y	Ceramic MS	15 ± 10	Multiple injections		20	2 cm^2^	3 ml	185 MBq
Order 1996 [15]	47	Pancreas carcinoma	^32^P	CPP + MAA	0.6–1.3 + 10–90	Single infusion	CT	22	T1-T3	<4.5 ml	148–1110 MBq
Tian 1996 [13]	33	27 HCC 6 liver metastasis	^90^Y	Glass MS	0.6–1.3 + 10–90	Multiple injections	US	18	1.8–10.7 cm	0.1–0.3 ml Max 1.5 ml/session	370/4440 MBq
Westlin 1997 [16]	17	Pancreas carcinoma	^32^P	CPP + MAA	0.6–1.3 + 10–90	Single infusion	US	19	13 cm^3^ (3.1–37.5)	Max 25% tumor volume	1390–9000 Gy
DeNittes 1999 [17]	5	Pancreas carcinoma	^32^P	CPP + MAA	0.6–1.3 + 10–90	Single infusion	US	22		3–4.5 ml	1110 MBq
Firusian 1999 [18]	17	Various solid malignancies	^32^P	CPP	0.6–2	Single infusion	US		10–290 cm^3^	5–15 ml	<20 cm^3^: 74 MBq 20–40 cm^3^: 148 MBq 50–100 cm^3^: 222 MBq >100 cm^3^: 2–3 sessions 100–200 cm^3^: 370 MBq 200–300 cm^3^: 555 MBq
Montijo 2003 [19]	1	Pancreas carcinoma	^32^P	CPP + MAA	0.6–1.3	Single infusion					544 MBq
Alimi 2007 [20]	14	Secondary resistant H&N tumors	^32^P	CPP	0.6–1.3	Single infusion	US			5–15 ml	<20 cm^3^: 74 MBq 20–40 cm^3^: 148 MBq 41–50 cm^3^: 185 MBq 51–100 cm^3^: 296 MBq >100 cm^3^: in 2–5 sessions >100 cm^3^: 444 MBq
Goh 2007 [14]	8	HCC	^32^P	BioSilicon MS	30	Multiple injections	US/CT	18 outer 22 inner		7% of tumor volume	4 MBq/cm^3^ of tumor
Rosemurgery 2008 [21]	30 18 treated 12 control	Pancreas carcinoma	^32^P	CPP	0.6–1.3	Multiple Injections	CT		19.7 ± 10.5 cm^2^ 24.1 ± 16.8 cm^2^	25% of tumor volume	(18.5 MBq/g tissue max 740 MBq Median dose 1255.34 Gy

*HCC* hepatocellular carcinoma, ^*90*^ *Y* yttrium-90, ^*32*^ *P* phosphorus-32, *CPP* chromic phosphate particles, *MAA* macroaggregated albumin, *MS* microspheres, *US* ultrasound, *CT* computed tomography, *MBq* megabecquerel, *Gy* Gray

^a^Tumor size presented as TNM stage, mean ± SD, or median and/or range, cm: diameter, cm^2^: tumor cross-sectional area, cm^3^: volume

**Table 3:** Technical details of animal studies

Study	Number and type of animals	Tumor type	Location SC/orthotopic/spontaneous	Isotope	Particle	Particle size (μm)	Number of injections	Needle gauge	Tumor size	Amount of fluid	Amount of activity (MBq)
Nakhgevany 1988 [22]	150 Lewis Wistar rats	Rat mammary carcinoma: AC33	SC	^90^Y	MS	18	1		0.73	0.5 ml	37
Brown 1991 [23]	6 BALB/c mice	Human mammary carcinoma: BT-20	SC	^166^Ho	Glass fragments	2–5 Irregular	1		19 (7–37) mm^3^	0.04 ml	7.4
Order 1994 [40]	27 Male ACI rats	Rat hepatoma: H4_2_E	SC	^32^P	CPP + MAA	0.6–1.3 + 10–90	1		0.5–1.5 mm		3.7
Lee 1997 [38]	C3hf/sed mice Nude mice Rats	Murine fibrosarcoma: FsaII Human colon carcinoma: LS174t Rat hepatoma: H4_2_E	SC	^32^P	CPP + MAA		1		FsaII /LS174t 0.5 cm^3^ H4IIE 1.5 cm^3^	0.01 ml HBSS/MAA	3.7, 7.4, 14.8
Nguyen 1997 [39]	Nude mice	Human melanoma: HBL Human head and neck squamous cell carcinoma: SCC1	SC	^32^P	CPP + MAA	0.6–1.3 + 10–90	1		1–1.5 cm^3^	0.1 ml MAA/^32^P	1.85
Watanabe 1997 [43]	Balb/c nude mice	Human neuroblastoma cell line: SK-N-MC	SC	^90^Y	MAA		1	Fine	1.0 cm^3^	0.05 ml	3.7
Zubillaga 1997 [9]	89 SD rats	NMU-induced breast carcinoma	Orthotopic	^32^P	CPP	2.5–4	1			0.05 ml	18.5
Wang 1998 [24]	42 SD rats	Rat hepatoma: N1S1	Orthotopic	^188^Re	Resin-MS	15 ± 2	1		2 cm	0.1 ml	7.4, 37
Zubillaga 1998 [8]	70 SD rats	NMU-induced breast carcinoma	Orthotopic	^32^P	CPP with charcoal	2.5–4	1			0.05 ml	18.5
Junfeng 1999 [41]	Kunming mice	Mice sarcoma: S180	SC	^188^Re	Sulfide suspension	1–5	1		1 cm	0.1 ml	17.02 + 6 days 23.31
Lee 1999 [37]	Nude mice	Human pancreatic carcinoma: AsPC-1	SC	^32^P	CPP + MAA	0.6–1.3 + 10–60	1		500 mm^3^	0.1 ml	
Lee I 1999 [36]	Nude mice	Human pancreatic carcinoma: AsPC-1 Human colon carcinoma: LS174t	SC	^32^P	CPP + MAA	0.6–4	1		500 mm^3^	0.1 ml	
Liu L 1999 [25]	Balb/c mice	Human liver cancer: H-CS	SC	^32^P	Glass MS	46–76	1		0.7–1.0 cm		183–7320 Gy
Junfeng 2000 [42]	Athymic nude mice	Human liver cancer: SMMC 7721	SC	^188^Re	Sulfide suspension	1–10	1		0.9–1.2 cm	0.1 ml	HBSS, 0, 3.7, 7.4, 18.5, 29.6 Repeated day 6
Lin 2000 [26]	SD rats	Rat hepatoma: N1S1	Orthotopic	^90^Y	Glass MS Theraspheres™	20–30	1		2 cm	0.1 ml	37
Chen 2001 [27]	SD rats	Rat hepatoma: N1S1	Orthotopic	^90^Y	Glass MS Theraspheres™	20–30	1		2 cm	0.1 ml	7.4
Lin 2005 [29]	NZW rabbits	Rabbit SCC: VX2	Orthotopic	^188^Re	Resin MS	15 ± 2	1	22	2–3 cm	2 ml	370
Zhang 2005 [28]	BALB/c mice	Human liver carcinoma: HepG2 human liver carcinoma: 2119	SC	^32^P	BioSilicon MS	20	1		65.3–88.9 mm^3^	50 μl	0.5, 1, 2
Hafeli 2007 [30]	SD rats	Rat gliosarcoma: 9L	Orthotopic	^186^Re ^188^Re	Glass MS	25–35	1		Identical location	2 × 10 μl Fibrin glue	1.85 ^188^Re/^186^Re (ratio 3:1)
Lubolt 2009 [35]	Wistar rats	Rat mammary carcinoma: Walker carcinoma 256 Rat Yoshida sarcoma	SC	^186^Re ^188^Re	Colloids MS	0.3 25			10–15 mm		
Bult 2012 [31]	NZW rabbits	Rabbit SCC: VX2	Orthotopic	^166^Ho	Acetylacetonate MS	15	1	29	2 cm^3^	0.1 ml	50
Bult 2013 [32]	24 Balb/C mice	Mice renal cell carcinoma	Orthotopic	^166^Ho	Acetylacetonate MS	10–15	1	29	5.6 ± 1.6 mm	0.01 ml	5
Bult 2013 [5]	3 DS cats	Various	Spontaneous liver	^166^Ho	Acetylacetonate MS	8 ± 2	Multiple injections	22	94–648 cm^3^		550–2170
Li 2014 [33]	Nude BABL/c mice	Human breast: MCF-7	SC	^131^I	Gelatin MS	30–50	1	27	0.83 cm^3^	0.1 ml 25% glucose	14.8, 92.5
Chi 2014 [34]	Nude BABL/c mice	Human HCC: HepG2	SC	^131^I	Gelatin MS	30–50	1	24	510 mm^3^	0.1 ml 25% glucose	7.4, 37

*DS* domestic shorthair, *SCC* squamous cell carcinoma, *HCC* hepatocellular carcinoma cell, *CPP* chromic phosphate particles, *MAA* macroaggregated albumin, *MS* microspheres. Tumor size: cm = diameter, cm^2^ = tumor cross-sectional area, cm^3^ = volume, *NMU N*-nitroso-*N*-methylurea

The MS were initially made of inert materials such as ceramics/glass, acetylacetonate [5, 31, 32], resin [24, 29], and plastics [10–13]. Nowadays, a large variety of biodegradable MS exists made of biosilicon [14, 28] and gelatin [33, 34]. The currently used MS are often chemically stable for at least the time that they remain radioactive, about 5–10 times the half-life of the incorporated isotope [14]. Thus, the minimum stability depends on the radioisotope; e.g., ^90^Y MS with a half-life of 2.6 days must be stable for at least 13 days and ^166^Ho with a half-life of 1.1 days at least 5.5 days. In most studies, stability was much longer than minimally required [31, 34].

The second group consisted of CPP with phosphorus-32 (^32^P). These particles were mostly used in the treatment of hemophilic arthropathy or natural cavities with malignant effusion. The main reason for a direct IT approach with these particles was the inability to deliver sufficient absorbed doses with systemic radioimmunotherapy [40]. In addition to the ^32^P CPP, ^188^Re sulfide particles were fabricated, with the advantage of easier production by generator and the possibility of SPECT imaging of the gamma radiation [42].

### Particle size

**Fig. 3:**
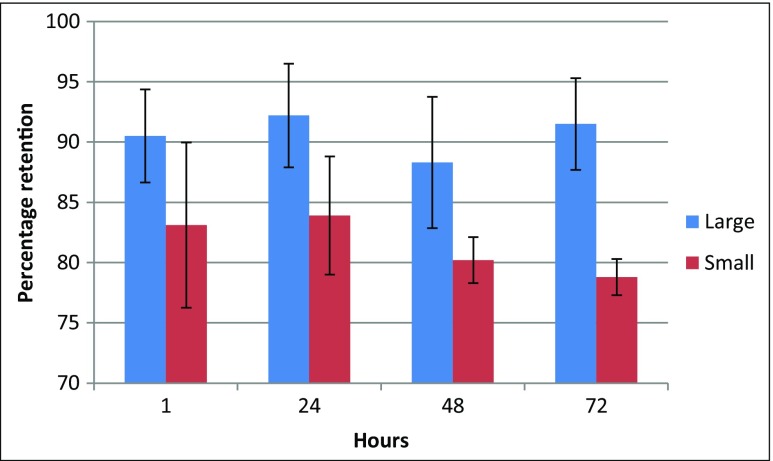
Intratumoral retention of rhenium-188 sulfide particles in Kunming mice with a sarcoma-180 tumor with a diameter of 1 cm. The larger particles (mix, 70.1% 1–5 μm; 19.8% 5–10 μm) showed a larger retention compared to the smaller particles (mix, 86.6% 1–5 μm; 10.9% 5–10 μm) [41]

In the included studies (see Tables 1 and 2), different particle sizes were used. Only one study investigated the preferred microparticle size for IT ablation [41]. In that study, two suspensions of ^188^Re sulfide particles with a particle size distribution of 70.1% of 1–5 μm and 19.8% of 5–10 μm particles compared to 86.6 and 10.9%, respectively, were injected in a sarcoma model with a diameter of 1 cm in Kunming mice [41]. The IT retention was higher for the larger particles at various time points (Fig. 3). A similar trend was observed in other studies that investigated the kinetics of IT injected microparticles compared to sub-micron [35], nanoparticles [9], or the effect of the addition of larger particles [40].

The effect of particle size on distribution, retention, elimination, and efficacy was clearly displayed in a study of five different sized phosphorus-32 (^32^P) compounds in 89 Sprague-Dawley rats with chemically induced breast tumors [9]. Thirty-two days after injection, an IT retention of radioactivity was found of 2.51 ± 0.39% for molecular ^32^P sodium orthophosphate (<1 nm), while 10–30 nm CPP had a retention of 28.93 ± 1.30%. The retention further increased for 30–70 nm (49.82 ± 5.41%) and 0.6–1.3 μm (51.61 ± 5.82%) sized particles. Larger charcoal CPP of 2.5–4.0 μm had the best retention of 84.50 ± 2.50% after 32 days. The elimination was primarily through urine and feces and had an inverse relationship with particle size ranging from 85.90 to 12.70% of the injected dose, respectively. The anti-tumor efficacy improved with higher retention because the tumor size ratios (tumor diameter after 32 days/tumor diameter at the start) after 32 days were 4.9 in non-treated controls and 4.5, 1.4, 1.1, 0.9, and 0.6 for the treated tumors in increasing order of particle size.

### Beta-emitting isotopes

Eight human studies used ^32^P and four used ^90^Y. These isotopes were often considered ideal by the authors because of their pure beta-emission. In animal studies, ^131^I, ^166^Ho, ^186^Re, and ^188^Re were used [44]. These isotopes also emit gamma radiation, which can be used for particle localization and quantitative imaging. None of the reviewed studies compared safety and efficacy between different radionuclides. Experience, production, biodistribution, imaging possibilities, pharmacokinetics, and clearance mostly defined preference [33, 42]. Besides these differences, relatively small differences in the energy spectrum, penetration depth, and half-life time exist. See Table 1.

### Technique

Due to the experimental nature of IT microbrachytherapy, no generally accepted standard administration method exists. Furthermore, most research was performed in small rodent tumor models, which are less informative for translation of the administration technique to the human situation. Therefore, the differences and similarities of the 12 reviewed human studies and the one treatment in veterinary patients will be described in the following paragraphs. These include differences in administration method, the amount of activity, the volume of injection, and imaging during and after administration.

### Administration methods

The larger MS were most often injected using multiple manual injection locations (i.e., sub-milliliter volumes) in a grid-like pattern [13, 21]. The smaller CPP were also administered through a single infusion technique in which a larger volume up to 4.5 ml of ^32^P CPP was administered in the tumor center, assuming that the pressure force would distribute the particles throughout the tumor [15]. Empirically, 5 cm was the largest tissue diameter satisfactorily covered by microparticles after a single infusion.

The specific characteristics of the needle used for IT injection were not frequently described. Needle sizes between 18 and 22G (outer diameter 1.2–0.7 mm) were commonly used percutaneously. In addition, an endoscopic ultrasound approach with a 22G needle was utilized in a published abstract [45] and unpublished trial (clinicaltrial.gov NCT00346281). Only a single study in eight humans with pancreatic tumors describes the use of a gel foam, which was injected through the 18G introducer needle to minimize back leakage and seal the needle tract [14].

### Volume of injection

No studies related the injected volume of fluid-suspended microparticles to the amount of leakage or distribution. The ideal amount of injected fluid to obtain the desired IT distribution is unknown. However, some suggested that larger fluid volumes might result in leakage of microparticles out of the tumor. With a higher amount of volume (i.e., 4.5 ml), high resistance with a sudden release of syringe pressure was often felt during infusion [15]. Subsequently, radioactivity was detected outside the tumor, presumably due to tissue destruction and leakage to surrounding tissues [15].

The amount of injection volume varied from 7 to 25% of the tumor volume in the most recent studies in pancreatic cancer [14, 21]. In liver tumors with a diameter of 2 to 8.8 cm, small (0.1–0.3 ml) volumes were used per location with a total maximum of 1.0–1.5 ml per treatment session [13]. Results obtained from non-particle intratumoral radionuclide therapies showed that larger volumes were associated with more side effects [46, 47]. In prostate cancer, 20–50 ml (equal to the prostate volume) was injected, which resulted in 55 adverse events like strictures, fistulas, and ulcers in the first 100 patients. In the subsequent 87 patients, only 2–3 ml injection fluid was used, and only eight events occurred. [46].

### Amount of activity/absorbed dose

The absorbed dose in tissue (Gray) varied from 120 to 19,300 Gy. A proper rationale for the injected activity or the desired absorbed dose was often missing. In a phase I study of ^32^P CPP in 28 patients with unresectable pancreas tumors, a maximum of 1110 MBq for a single infusion was decided [15]. This empirically determined maximum was based on the expected limitation of the injection volume of 4.5 ml. This approach resulted in a maximum cumulative absorbed dose of 17,000 Gy. A more accurate dosing approach was applied in recent studies on pancreas cancer patients. However, the injected activity per gram (or cm^3^) in the RCT with ^32^P CPP and the cohort study with ^32^P BioSilicon MS still varied with a factor of 4.6 (4 vs. 18.5 MBq/cm^3^ tumor) [14, 21].

### Image-guided administration

The administration procedure was image guided in nine human studies. In most animal studies (*n* = 22), no imaging was used during the administration. In the veterinary patients and rabbit studies with liver tumors, ultrasound guidance (*n* = 2) was used, and a stereotactic frame was used in glioma-bearing rats. With CT or ultrasound, the tip of the needle was positioned at the desired location before administration. Some authors preferred ultrasound because this modality provided easy and real-time imaging during the actual injection [13]. During the injections of MS, echogenic spots were sometimes seen “flowing” in some narrow, vessel-like gaps and sometimes even out of the tumor boundaries, especially after a fast, forceful injection [13]. Subsequently, shaking of the vial before administration, resulting in air bubbles, was used to visualize any major unexpected leakage outside the tumor during the injection on ultrasound [16].

### Outcomes

The primary outcomes of IT microbrachytherapy were safety and efficacy. However, a more fundamental understanding of this treatment is necessary, especially because the outcomes of safety and efficacy probably mostly depend on the distribution of the activity. Therefore, distributional data will be described first.

### Distribution

Insufficient retention of radioactive microparticles leads to an insufficiently absorbed dose and therefore an ineffective treatment. However, apart from total absorbed dose, the IT distribution of activity throughout the tumor is crucial, as “missed” parts of the tumor will result in residual vital tumor. Leakage of activity, on the other hand, may lead to an unintended absorbed dose to healthy tissue and could potentially result in side effects.

### Leakage

Several potential routes of leakage were identified, and a distinction was made between external leakage and internal leakage. External leakage from the syringe occurred twice during treatment of pancreatic tumors with ^32^P CPP infusion due to high resistance in the tumor [16]. The authors experienced in an experiment, with ^166^Holmium microsphere injections in ex vivo tissues, a needle disconnection from a Luer lock syringe after exerting high pressure to overcome tissue resistance. Another possible route of external leakage is injection canal leakage. Injection canal leakage was not described in the human or animal studies. However, in the study of eight humans with pancreatic tumors, the authors describe the use of a gel foam pledget/slurry which was injected through the introducer needle to minimize back leakage and seal the needle tract [14].

Internal leakage to non-target tissues was divided in hematogenous or intravenous and intraductal leakage. In the majority of human [13, 15, 16] and animal [8, 9, 24, 35, 39, 43] studies, some degree of intravenous leakage or shunting of particles through the capillary bed was described. After the improved retention of CCP particles with an additional injection of larger 10–90 μm MAA particles, 56 vs. 90%, respectively, and the hypothesis of a vascular blockade, vascularity became an important variable for leakage. ^32^P CCP 0.6–1.3 μm was injected in nude mice with human pigmented melanoma cell line (HBL) and a human squamous cell carcinoma cell line (SCC1); three to four times higher organ counting was found in SCC1 [39]. This phenomenon is probably explained by the difference in vascularity between HBL and SCC1 tumors, which contained 5.7 vs. 21.4 blood vessels/mm^2^, respectively.

In Wistar rats, ^188^Re MS (25 μm) and small ^186^Re sulfide particles (0.3 μm) were injected in hypervascularized Walker 256 carcinomas and hypovascularized Yoshida sarcomas. This study revealed a bi-phasic drainage of the injected particles out of the tumor. A fast wash-out phase, where the IT activity decreases to approximately 70% within 10 min, was followed by a slow decline in which IT activity falls to 60% of the initially injected activity at 48 h. The fast leakage was more pronounced in hypervascularized tumors with smaller particles, whereas the slow decline was independent of particle size and vascularity [35].

In addition, the distribution of activity after IV leakage depends on the tumor location and particle size. In 33 liver cancer patients treated with ^90^Y MS, a lung shunt of 9–20% of the injected activity was observed in six patients [13]. Similar shunts were observed after IT injections with ^188^Re MS in rats with subcutaneous and liver tumors which resulted in trapped MS in the pulmonary capillary bed [24, 35]. Detected activity in the liver, after the treatment of the pancreas, was probably caused by venous shunting of CCP + MAA [15, 16]. However, small particles such as CCP (±1 μm) and ^186^Re-sulfide particles (0.3 μm) can probably also pass through the capillary bed of the tumor and phagocytized in the reticuloendothelial system and therefore detected in the liver.

During the treatment of malignancies in the pancreas and liver, intraductal leakage and activity in the gastrointestinal tract were described. In the 48 patients with ^32^P CCP infusion in pancreatic cancer, accidental needle placement and injection into the pancreatic duct occurred. Forty-eight hours after injection, all intestinal activity was excreted without gastrointestinal toxicity [15]. During the treatment of 33 patients with liver malignancies with ^90^Y MS, a similar leakage was found in the intestines in four patients that disappeared within 1–2 days [13].

Lymphatic drainage is an additional potential route which was however not observed in the reviewed studies. This well-known route of tumor drainage is commonly used in the sentinel node procedure. The microparticles were presumably too large for drainage of significant amounts of radioactivity to the draining lymph nodes.

### Safety

The safety and toxicity were closely related to the distribution. The safety or clinical complications were divided into local and systemic side effects. The experimental treatment was often performed in progressively ill patients [10]. The probability of a causal relationship between an event and treatment was therefore often difficult to determine. However, in general, the authors of both animal and human studies concluded that the treatment was safe.

A safety concern, which was not described in the clinical studies, was needle tract metastasis. This complication might have occurred in one animal study. After three thallium-201 injections in eight Fischer 344 rats with an orthotopic glioma model, five metastases occurred of which three were along the needle tract [48]. Whether this was due to disruption of natural barriers or by dragging cells into the needle tract was ambiguous.

### Local side effects

A reported local side effect in eight pancreatic tumor patients treated with ^32^P BioSilicon MS was pain at the injection site (*n* = 3) and the treated region (*n* = 1) which resolved within 1 or 2 days [14]. Similar results were found with ^32^P CPP in the pancreas. The injection of ^90^Y MS in the liver was not painful, in contrast to ethanol injections [13]. Another mild effect that was observed twice was transient erythema after microbrachytherapy of superficial cervical lymph node metastasis of H&N tumors with ^32^P CPP [18]. In the 23 patients from the three case series treated with ^90^Y MS, the following four local complications were reported: a rectovesical fistula in prostate cancer, a lung abscess and localized radiation fibrosis in bronchial cancer, and a skin defect in a rhabdomyosarcoma of the nose [10–12, 14]. In addition, after treatment of pancreas cancer with ^32^P, some patients had increased serum amylase as a sign of local damage [14, 15, 21].

In the randomized trial of 30 patients with pancreas carcinoma treated with a combination of 5FU, 60 Gy EBRT, and gemcitabine [21], 18 patients were additionally treated with ^32^P therapy. A gastrointestinal bleeding was experienced in 15 patients of whom 13 were treated with ^32^P. In eight patients, this complication seemed attributable to pancreatic tumor eroding into the duodenum. This complication was described in two other pancreas carcinoma patients treated with ^32^P CPP [16, 19] (Table 4).

**Table 4:** Outcomes of distribution, efficacy, and safety of human studies

Study	Number of patients	Tumor type	Isotope	Retention	Leakage	Toxicity	Efficacy
Kim 1962 [10]	10	Breast, bladder, brain prostate, lung, metastasis	^90^Y			1 localized radiation fibrosis	*N* = 4 regression *N* = 2 no response *N* = 3 no data *N* = 1 died before evaluation
Blanchard 1965 [11]	12	Bladder, prostate, breast, lung, and metastasis	^90^Y			1 lung abscess 1 rectovesical fistula 1 pancytopenia	*N* = 1 marked regression *N* = 2 regression *N* = 2 no regression *N* = 7 no data
Ariel 1978 [12]	1	Rhabdomyosarcoma	^90^Y			1 skin defect	*N* = 1 complete response
Order 1996 [15]	47	Pancreas carcinoma	^32^P	Patients without metastasis Without shunting *N* = 15 patients Mean 96% (range 86–100%) With shunting to the liver *N* = 12 patients Mean 52% (range 17–88%) With metastasis *N* = 19 patients Mean 79% (range 22–100%)	Blood 1.85–3552 Bq/ml *N* = 12 shunting to the liver	Without metastases 2 Gr III leukopenia 2 Gr IV thrombocytopenia 1 Gr III amylase 1 Gr IV amylase With metastases 1 Gr III leukopenia 1 Gr III thrombocytopenia 2 Gr IV thrombocytopenia	*N* = 7 complete response *N* = 11 partial response
Tian 1996 [13]	33	27 HCC 6 liver metastasis	^90^Y	Biological T^1/2^ = 57.6 ± 1.02 h Physical T^1/2^ = 66 h	Most patients liver (outside tumor) 3.1–11.6% *N* = 6 lung 8.8–20.8% *N* = 4 intestines	1 acute myocardial infarction day 0 of 2nd treatment 2 temp leukopenia after combination with chemotherapy	Tumor shrinkage rate^a^ *N* = 12 ≥50% or more *N* = 12 25–50% *N* = 5 ≤25% *N* = 3 no change *N* = 6 no data
Westlin 1997 [16]	17	Pancreas carcinoma	^32^P		*N* = 2 intestines *N* = 2 liver	1 arterial bleeding 2 slightly decreased blood counts	*N* = 4 complete response *N* = 5 partial response *N* = 7 stable disease *N* = 1 no data
DeNittes 1999 [17]	5	Pancreas carcinoma	^32^P	100%		No significant toxicity	*N* = 2 complete response *N* = 3 stable disease
Firusian 1999 [18]	17	Various solid malignancies	^32^P	Biologic T^1/2^ = physical T^1/2^	Blood <11 Bq/ml	1 Gr IV thrombocytopenia 2 erythema of skin after superficial LN treatment	*N* = 7 complete response *N* = 5 partial response *N* = 5 no response
Montijo 2003 [19]	1	Pancreas carcinoma	^32^P			Arterial bleeding fistula	*N* = 1 died before evaluation
Alimi 2007 [20]	14	Secondary resistant H&N tumors	^32^P	Biologic T^1/2^ = physical T^1/2^		3 Gr I/II thrombocytopenia 2 transient erythema	*N* = 8 partial response *N* = 6 no response
Goh 2007 [14]	8	HCC	^32^P		No detectable radioactivity in blood samples	3 injection site pain 2 fatigue 2 portal hypertension 1 abdominal pain 1 rigors 1 vomiting 1 Gr IV diabetes mellitus 1 Gr III neutropenia 1 Gr III pancytopenia	12 weeks *N* = 2 complete response *N* = 2 partial response *N* = 4 stable disease 24 weeks *N* = 2 complete response *N* = 2 partial response *N* = 1 progressive disease *N* = 3 withdrawn
Rosemurgery 2008 [21]	30 18 treated 12 control	Pancreas	^32^P		Intestines	^32^P/Co SAEs 75/22 Hospitalizations 34/10 GI bleedings 13/2 Pancytopenia 1/0 Leukocytopenia 1/0 Anemia 5/4 Thrombocytopenia 5/0	Survival ^32^P 5.2 months Co 12.2 months Tumor size Prior → post-treatment ^32^P 16.1 → 13.3 cm^2^ Co 20.0 → 12.4 cm^2^

^*90*^ *Y* yttrium-90, ^*32*^ *P* phosphorus-32, cm^2^: tumor cross-sectional area, *SAE* serious adverse events, *Gr* grade, *GI* gastrointestinal

^a^ *N* number of lesions in 27/33 patients

### Systemic side effects

Hematological abnormalities were a frequently described side effect. This could result from treatment of blood-pooled organs, leaking of activity from microspheres, or disintegration of microspheres into smaller particles. Most of the used radioactive isotopes do have an increased accumulation in bone after leakage, which may result in bone marrow suppression. Pancytopenia was described in 1965 in a patient in whom 10% of the activity leaked from an inadequate batch of ^90^Y MS. In the cohort of 48 pancreas carcinoma patients, grade 3 leukopenia and grade 3/4 thrombocytopenia were observed in three and five patients, respectively [15]. Additionally, after treatment of the liver with ^90^Y MS, leukopenia was observed in 2 out of 33 patients [13]. However, since the amounts of activity were low (venous samples <11 Bq/ml) [18], the leakage often did not result in clinical toxicity.

### Efficacy

The tumoricidal efficacy of intratumoral treatment with radioactive beta-emitting microparticles was shown in animal models. Forty nude mice with subcutaneous liver tumors were treated with ^32^P glass MS. This study did not only show that ^32^P glass MS were effective in the treatment of a subcutaneous liver tumor model; it additionally showed a dose-response relation [25]. The tumor-inhibiting rate improved from the lowest dose of 183 Gy to the highest dose of 7320 Gy, from 59.7 to 93.6%, respectively. These results were confirmed in another liver carcinoma line in nude mice with ^188^Re [42] (Table 5).

**Table 5:**
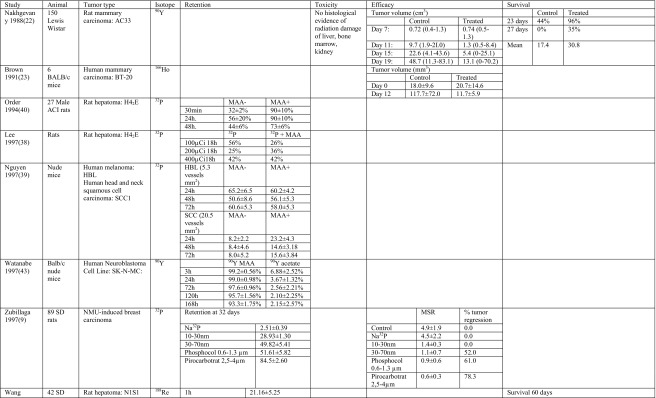

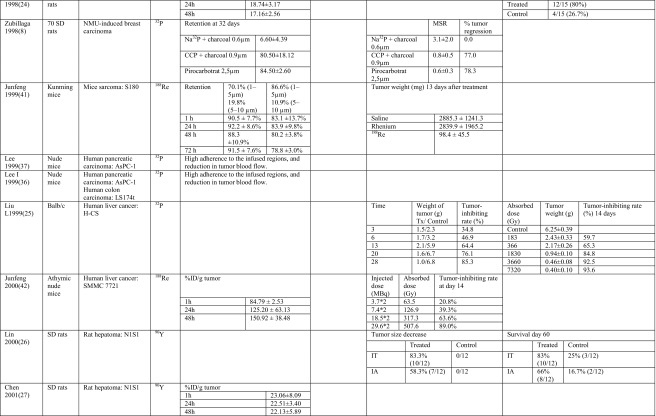

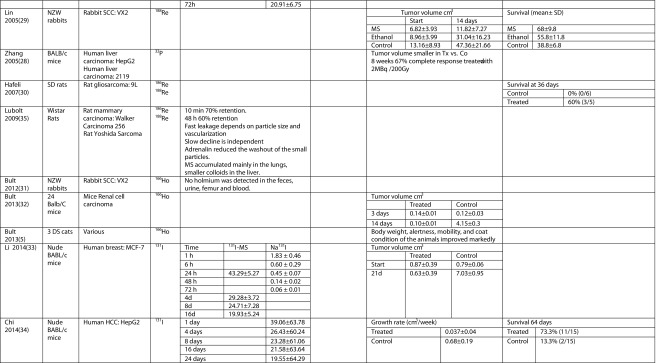
Main outcomes of animal studies

The efficacy in the human studies was more difficult to interpret as 11/12 were non-comparative studies (Table 4). However, the results of eight patients with pancreas carcinomas treated with ^32^P BioSilicon MS were promising, with two complete responses, two partial responses, and four patients with stable disease after 12 weeks [14]. Furthermore, a survival benefit was found in the responders as compared to the non-responders for 14 head and neck cancer patients treated with ^32^P CCP. On the other hand, a survival benefit was not found in the RCT in 30 pancreas cancer patients with a treatment history of 5-FU, EBRT, and gemcitabine. Patients receiving ^32^P CCP in addition to standard therapy survived a median of 5.2 months, whereas patients receiving standard therapy alone survived 12.2 months, *p* = 0.16. A decrease in radiologic tumor size was not detected on CT because cancer persisted along the periphery of the injection sites [21].

## Discussion

In this study, all currently available literature on the potential role of beta-emitting microparticles for IT treatment of solid malignant neoplasms was reviewed. The results of 12 human and 25 animal studies were included. The large variety of particles, techniques, and treated tumors in the studies provided an important insight into issues concerning efficacy, safety, particle and isotope choice, and other concepts for future research.

Is microbrachytherapy effective? Based on the reviewed data, it can be concluded that beta-emitting microparticles seem to be an effective tumoricidal agent. The majority of the studies showed promising results in both humans and animals with complete responses and long-term survival [14]. However, a direct IT injection with tumoricidal particles does not automatically lead to an effective tumor treatment [21]. Obtaining a sufficient dose coverage of all tumor tissue requires the challenging design of an optimal treatment modality with regard to biological stability, injection techniques, dosimetry, biodistribution, etc.

Is microbrachytherapy safe? In the only performed RCT, concerns were raised about the safety of additional IT treatment with small ^32^P CPP in pancreas cancer patients treated with 5-fluorouracil, EBRT, and gemcitabine [21]. More patients experienced gastrointestinal bleeding compared to the standard therapy alone. Bleedings were not observed in studies with other particles and other tumors. Other local side effects included manageable discomfort at the injection site. Except for manageable hematological abnormalities, other systemic adverse events were not encountered. Therefore, apart from pancreas tumors, IT treatment seems to be a reasonably safe alternative.

Can we predict complications? Leakage appears to follow the path of least resistance. An easy route of leakage after IT administration is injection canal leakage. The use of a small needle can reduce this. However, care should be taken to prevent premature settling and clotting of microparticles inside the syringe and blocking the needle [5, 31, 32]. A 21G needle seems to be the preferred needle to use. Additional measures to reduce leakage may include slow injection and withdrawal of the needle with slight pressure or injection of obstructing pledget/foam. Other routes of leakage (i.e., intravascular or intraductal) may be caused by injection position, excessive volume, or pressure. Increased permeability of tumor neovascularization may be considered a risk factor for hematogenous leakage. Leakage of an entire dose may happen when a single infusion technique is used [15]. A grid-like injection procedure with larger MS in small volume depots may, therefore, be preferred over the infusion of smaller particles.

How much fluid should be injected during microbrachytherapy? Theoretically, more fluid results in more propelling force and a more homogeneous distribution of microparticles in the target tissue. This should be balanced against the chances of more side effects [46, 47]. The injected volume should probably range between 7 and 30% of the tumor volume [14, 16] as excessive volume or pressure may result in leakage [15, 16]. In addition, intratumoral pressure depends on tumor characteristics and location and should be taken into account [36–38]. A more viscous fluid may be used to obtain even more control [6, 12]. For example, 25% glucose, fibrin glue, and other formulas were used to improve the injection procedure [14, 30], or hydrogels such as chitosan [49].

Which particles should be used? There is a relation between particle size and retention: the larger the particle, the higher the retention. Subsequently, preferences for the larger MS exist. On the other hand, particles must be small enough to distribute evenly throughout the tumor to deliver an adequate homogeneously absorbed dose. The optimal number of particles was not mentioned in the studies, but it is likely to influence biodistribution, safety, and efficacy too, and must be investigated to result in a better understanding of IT injection.

What are the ideal radionuclide characteristics? ^90^Y is often considered the ideal isotope, with a high energy, pure beta-emitter for easy radiation protection, and an intermediate half-life of 64 h. However, because of the questions related to both IT distribution and retention of microparticles, isotopes with better imaging properties are more suitable for imaging-guided monitoring of IT particle distribution and dosimetry. For leakage to other organs, low-resolution bremsstrahlung scintigraphy is sufficient. However, the resolution of this technique is insufficient for local tumor dose distribution monitoring. SPECT imaging can greatly improve particle distribution measurements for ^186^Re, ^188^Re, and ^166^Ho because of the associated gamma-radiation of 80–200 keV. Furthermore, ^166^Ho can be visualized and quantified with CT and MRI [32]. There are several developments in imaging of these isotopes. ^90^Y PET/CT is also quantitative but requires long acquisition times due to the low number of positrons. Another relative new imaging opportunity is Cerenkov luminescence imaging (CLI) [50]. CLI could provide quantitative high-resolution imaging and image-based dosimetry for a large variety of isotopes [50–52]. The main limitation of CLI is the limited penetration depth of light of approximately 10 mm into tissue, however very promising, in small animal models [51, 53].

In addition to imaging characteristics, half-life and beta energy should be considered in relation to efficacy and safety, but also logistics, like production and cost. In this respect, a generator like the tungsten-188/rhenium-188 generator may be beneficial. In theory, a high dose rate (i.e., short half-life) will prevent the recovery of radiation damaged tumor cells and may lead to higher efficacy. In terms of logistics, a short half-life may lead to production and logistic challenges on the one hand, but a shorter hospital stay with fewer restrictions after discharge on the other hand.

IT injections can be performed in a variety of tumor types and organs. Based on the postulated methods of leakage, potential risks of side effects, and more challenging administration, the pancreas seems to be a difficult-to-treat organ. Superficial tumors, such as lymph node metastases of the head and neck region, and liver tumors are better accessible, show minimal leakage, and have minimal side effects. With increasing knowledge, microbrachytherapy may be adjusted to tumor characteristics, for example, the addition of a vasoconstrictive drug in hypervascular tumors.

## Conclusion

Intratumoral treatment with radioactive beta-emitting microparticles, microbrachytherapy, in solid malignant neoplasms may have additional value for patients with tumors at various locations. The uncomplicated treatments with high cumulative doses of up to 19,000 Gy suggest that microbrachytherapy is relatively safe. Larger particles resulted in a higher retention and tumor-inhibiting efficacy of >90% with an intratumoral absorbed dose of 7320 Gy. A small injected volume of 7–30% of the tumor volume divided in small volume depots, 0.1–0.3 ml, administered in a grid-like injection procedure is preferred. With accurate administration and high-resolution imaging, the efficacy may be improved while the risk of side effects will be reduced. Particles that emit a small amount of gamma-radiation and can be visualized with high-resolution imaging are preferred at this stage. Experiments should be performed in larger tumor models to obtain better clinical relevant data on the IT distribution. Subsequently, the threshold absorbed dose to successfully treat the tumor should be investigated. Furthermore, accurate administration requires skilled physicians and controlled injection, and will be time consuming. In the near future, with advanced technologies such as controllable needle placement and injection systems, the procedure could be performed easily, quickly, and safely for patients and personnel.

## Appendix. Search strategy

### Medline

1. Humans [Mesh]
2. Animals [Mesh]
3. Animal [Title/Abstract]
4. Human [Title/Abstract]
5. #1 OR #2 OR #3 OR #4
6. Neoplasms [Mesh]
7. Tumor*[Title/Abstract]
8. Tumour*[Title/Abstract]
9. Cancer*[Title/Abstract]
10. #6 OR #7 OR #8 OR #9
11. Intratumor*[Title/Abstract]
12. Intra-tumor*[Title/Abstract]
13. Intratumour*[Title/Abstract]
14. Intra-tumour*[Title/Abstract]
15. Intralesion*[Title/Abstract]
16. Intra-lesion*[Title/Abstract]
17. Interstitial*[Title/Abstract]
18. #10 OR #11 OR #12 OR #13 OR #14 OR #15 OR #16 OR 17#
19. Radiotherapy [Mesh Terms]
20. Radiotherapy [Title/Abstract]
21. Radioisotopes [Mesh]
22. Isotopes [MeSH]
23. Gold [Mesh]
24. Gold [Title/Abstract]
25. Lutetium [Mesh]
26. Lutetium [Title/Abstract]
27. Rhenium [Mesh]
28. Rhenium [Title/Abstract]
29. Holmium [Mesh]
30. Holmium [Title/Abstract]
31. Iodine [Mesh]
32. Iodine [Title/Abstract]
33. Yttrium [Mesh]
34. Yttrium [Title/Abstract]
35. Phosphorus [Mesh]
36. Phosphorus [Title/Abstract]
37. 32P[Title/Abstract]
38. P32 [Title/Abstract]
39. 32-P [Title/Abstract]
40. P-32 [Title/Abstract]
41. #19 OR #20 OR #21 OR #22 OR #23 OR #24 OR #25 OR #26 OR #27 OR #28 OR #29 OR #30 OR #31 OR #32 OR #33 OR #34 OR #35 OR #36 OR #37 OR #38 OR #39 OR #40 OR #41
42. “Treatment Outcome”[Mesh]
43. Survival [Title/Abstract]
44. “Tissue Distribution”[Mesh]
45. Distribut*[Title/Abstract]
46. Safe*[Title/Abstract]
47. Toxicity [Subheading]
48. Toxic*[Title/Abstract]
49. Effic*[Title/Abstract]
50. Effec*[Title/Abstract]
51. #42 OR #43 OR #44 OR #45 OR #46 OR #47 OR #48 OR #49 OR #50
52. #5 AND #10 AND #18 AND #41 AND #51

### EMBASE

1. ‘in vivo study’/exp
2. ‘human’/exp
3. ‘animal’/exp
4. #1 OR #2 OR #3
5. ‘neoplasm’/exp
6. cancer:ab,ti
7. tumo*r:ab,ti
8. #5 OR #6 OR #7
9. ‘intratumoral drug administration’/exp
10. intratumo*r*:ab,ti
11. intralesion*:ab,ti
12. ‘interstitial’:ab,ti
13. ‘intra tumo*r*’:ab,ti
14. ‘intra lesion*’:ab,ti
15. #9 OR #10 OR #11 OR #12 OR #13 OR #14
16. ‘radiotherapy’/exp
17. ‘radiotherapy’:ab,ti
18. ‘radioisotope’/exp
19. ‘radioisotope’:ab,ti
20. ‘isotope’/exp
21. ‘isotope’:ab,ti
22. ‘gold’/exp
23. ‘gold’:ab,ti
24. ‘lutetium’/exp
25. ‘lutetium’:ab,ti
26. ‘rhenium’:ab,ti
27. ‘rhenium’/exp
28. ‘holmium’/exp
29. ‘holmium’:ab,ti
30. ‘iodine’:ab,ti
31. ‘iodine’/exp
32. ‘yttrium’/exp
33. yttrium:ab,ti
34. ‘phosphorus’/exp
35. ‘phosphorus’:ab,ti
36. p32:ab,ti
37. ‘p 32’:ab,ti
38. ‘32p’:ab,ti
39. ‘32 p’:ab,ti
40. ‘phosphorus 32’:ab,ti
41. #15 OR #16 OR #17 OR #18 OR #19 OR #20 OR #21 OR #22 OR #23 OR #24 OR #25 OR #26 OR #27 OR #28 OR #29 OR #30 OR #31 OR #32 OR #33 OR #34 OR #35 OR #36 OR #37 OR #38 OR #39
42. ‘treatment outcome’/exp
43. ‘survival’/exp
44. survival:ab,ti
45. ‘toxicity’/exp
46. tox*:ab,ti
47. ‘safety’/exp
48. safety:ab,ti
49. ‘tissue distribution’/exp
50. distribution:ab,ti
51. effic*:ab,ti
52. effec*:ab,ti
53. #42 OR #43 OR #44 OR #45 OR #46 OR #47 OR #48 OR #49 OR #50 OR #51 OR #52
54. #4 AND #8 AND #15 AND #41 AND #53

### Central

1. MeSH descriptor: [Humans] explode all trees
2. MeSH descriptor: [Animals] explode all trees
3. in-vivo:ti,ab,kw or human:ti,ab,kw or animal:ti,ab,kw
4. #1 or #2 or #3
5. MeSH descriptor: [Neoplasms] explode all trees
6. cancer:ti,ab,kw
7. neoplasia:ti,ab,kw
8. neoplasm:ti,ab,kw
9. tumor:ti,ab,kw
10. tumour:ti,ab,kw
11. #5 or #6 or #7 or #8 or #9 or #10
12. intratumor*:ti,ab,kw
13. intratumour*:ti,ab,kw
14. intralesion*:ti,ab,kw
15. intra-tumor*:ti,ab,kw
16. intra-tumour*:ti,ab,kw
17. intra-lesion*:ti,ab,kw
18. interstitial:ti,ab,kw
19. #12 or #13 or #14 or #15 or #16 or #17 or #18
20. MeSH descriptor: [Radiotherapy] explode all trees
21. Radiotherapy:ti,ab,kw
22. MeSH descriptor: [Radioisotopes] explode all trees
23. radioisotope*:ti,ab,kw
24. MeSH descriptor: [Isotopes] explode all trees
25. phosphorus*:ti,ab,kw
26. yttrium:ti,ab,kw
27. Iodine:ti,ab,kw
28. Holmium:ti,ab,kw
29. Lutetium:ti,ab,kw
30. Rhenium:ti,ab,kw
31. Gold:ti,ab,kw
32. #20 or #21 or #22 or #23 or #24 or #25 or #26 or #27 or #28 or #29 or #30 or #31
33. MeSH descriptor: [Treatment Outcome] explode all trees
34. MeSH descriptor: [Safety] explode all trees
35. MeSH descriptor: [Tissue Distribution] explode all trees
36. effic*:ti,ab,kw or effec*:ti,ab,kw or Safety:ti,ab,kw or distribut*:ti,ab,kw or toxic*:ti,ab,kw
37. survival:ti,ab,kw
38. #33 or #34 or #35 or #36 or #37
39. #4 and #11 and #19 and #32 and #38

## References

[1] Lyon SM, Pascoe DM (2004). Percutaneous gastrostomy and gastrojejunostomy. Semin Intervent Radiol Thieme Medical Publishers.

[2] Loffroy R, Favelier S, Chevallier O, Estivalet L, Genson P-Y, Pottecher P (2015). Preoperative portal vein embolization in liver cancer: indications, techniques and outcomes. Quant Imaging Med Surg.

[3] de Baere T, Elias D, Dromain C, Din MGE, Kuoch V, Ducreux M (2000). Radiofrequency ablation of 100 hepatic metastases with a mean follow-up of more than 1 year. Am J Roentgenol American Roentgen Ray Society.

[4] Bussu F, Tagliaferri L, Mattiucci G, Parrilla C, Dinapoli N, Miccichè F (2016). Comparison of interstitial brachytherapy and surgery as primary treatments for nasal vestibule carcinomas. Laryngoscope.

[5] Bult W, Vente MADD, Vandermeulen E, Gielen I, Seevinck PR, Saunders J (2013). Microbrachytherapy using holmium-166 acetylacetonate microspheres: a pilot study in a spontaneous cancer animal model. Brachytherapy United States.

[6] Booth A, Clarke M, Dooley G, Ghersi D, Moher D, Petticrew M (2012). The nuts and bolts of PROSPERO:an international prospective register of systematic reviews. Syst Rev Springer Open Ltd.

[7] Stang A (2010). Critical evaluation of the Newcastle-Ottawa scale for the assessment of the quality of nonrandomized studies in meta-analyses. Eur J Epidemiol.

[8] Zubillaga MB, Boccio JR, Nicolini JO, Ughetti R, Lanari E, Caro RA (1998). Radiochemical and radiopharmacological properties of Pirocarbotrat and other labeled charcoal dispersions: comparative studies in rats with NMU-induced mammary tumors. Nucl Med Biol.

[9] Zubillaga MB, Boccio JR, Nicolini JO, Ughetti R, Lanari E, Caro R (1997). A. Pirocarbotrat: a new radiopharmaceutical for the treatment of solid tumors—comparative studies in N-nitrosomethylurea-induced rat mammary tumors. Nucl Med Biol ENGLAND.

[10] Kim YS, Lafave JW, Maclean LD (1962). The use of radiating microspheres in the treatment of experimental and human malignancy. Surgery.

[11] Blanchard RJ, Lafave JW, Kim YS, Frye CS, Ritchie WP, Perry JF (1965). Treatment of patients with advanced cancer utilizing Y90 microspheres. Cancer.

[12] Ariel IM (1978). Cure of an embryonal rhabdomyosarcoma of the nose of an infant by interstitial 90Yttrium microspheres: a case report. Int J Nucl Med Biol.

[13] Tian JH, Xu BX, Zhang JM, Dong BW, Liang P, Wang XD (1996). Ultrasound-guided internal radiotherapy using yttrium-90-glass microspheres for liver malignancies. J Nucl Med.

[14] Goh ASW, Chung AYF, Lo RHG, Lau TN, Yu SWK, Chng M (2007). A novel approach to brachytherapy in hepatocellular carcinoma using a phosphorus 32 (32P) brachytherapy delivery device-a first-in-man study. Int J Radiat Oncol Biol Phys.

[15] Order SE, Siegel JA, Principato R, Zeiger LE, Johnson E, Lang P et al Selective tumor irradiation by infusional brachytherapy in nonresectable pancreatic cancer: a phase I study. Int J Radiat Oncol Biol Phys 10.1016/s0360-3016(96)00484-1 8985034

[16] Westlin J-E, Andersson-Forsman C, Garske U, Linné T, Aas M, Glimelius B (1997). Objective responses after fractionated infusional brachytherapy of unresectable pancreatic adenocarcinomas. Cancer.

[17] DeNittis AS, Stambaugh MD, Lang P, Wallner PE, Lustig RA, Dillman RO (1999). Complete remission of nonresectable pancreatic cancer after infusional colloidal phosphorus-32 brachytherapy, external beam radiation therapy, and 5-fluorouracil: a preliminary report. Am J Clin Oncol Cancer Clin Trials.

[18] Firusian N, Dempke WCM (1999). An early phase II study of intratumoral P-32 chromic phosphate injection therapy for patients with refractory solid tumors and solitary metastases. Cancer.

[19] Montijo IJJ, Khurana V, Alazmi WM, Order SE, Barkin JS, Jaca Montijo IJ (2003). Vascular pancreatic gastric fistula: a complication of colloidal 32P injection for nonresectable pancreatic cancer. Dig Dis Sci.

[20] Alimi KA, Firusian N, Dempke W (2007). Effects of intralesional 32-P chromic phosphate in refractory patients with head and neck tumours. Anticancer Res.

[21] Rosemurgy A, Luzardo G, Cooper J, Bowers C, Zervos E, Bloomston M (2008). 32P as an adjunct to standard therapy for locally advanced unresectable pancreatic cancer: a randomized trial. J Gastrointest Surg.

[22] Nakhgevany KB, Mobini J, Bassett JG, Miller E (1988). Nonabsorbable radioactive material in the treatment of carcinomas by local injections. Cancer.

[23] Brown RF, Lindesmith LC, Day DE (1991). 166Holmium-containing glass for internal radiotherapy of tumors. Nucl Med Biol.

[24] Wang SJ, Lin WY, Chen MN, Chi CS, Chen JT, Ho WL (1998). Intratumoral injection of rhenium-188 microspheres into an animal model of hepatoma. J Nucl Med.

[25] Liu L, Jiang Z, Teng G-J, Song J-Z, Zhang D-S, Guo Q-M (1999). Clinical and experimental study on regional administration of phosphorus 32 glass microspheres in treating hepatic carcinoma. World J Gastroenterol United States.

[26] Lin WY, Tsai SC, Hsieh JF, Wang SJ (2000). Effects of 90Y-microspheres on liver tumors: comparison of intratumoral injection method and intra-arterial injection method. J Nucl Med.

[27] Chen SD, Hsieh JF, Tsai SC, Lin WY, Cheng KY, Wang SJ (2001). Intra-tumoural injection of 90Y microspheres into an animal model of hepatoma. Nucl Med Commun.

[28] Zhang K, Loong SLE, Connor S, Yu SWK, Tan SY, Ng RTH (2005). Complete tumor response following intratumoral 32P BioSilicon on human hepatocellular and pancreatic carcinoma xenografts in nude mice. Clin Cancer Res.

[29] Lin YC, Tsai SC, Hung GU, Lee JC, Huang YS, Lin WY (2005). Direct injection of 188Re-microspheres in the treatment of hepatocellular carcinoma. Compared with traditional percutaneous ethanol injection: an animal study. Nuklearmedizin.

[30] Haefeli UO, Pauer GJ, Unnithan J, Prayson RA (2007). Fibrin glue system for adjuvant brachytherapy of brain tumors with 188Re and 186Re-labeled microspheres. Eur J Pharm Biopharm.

[31] Bult W, De Leeuw H, Steinebach OM, Van Der Bom MJ, Wolterbeek HT, Heeren RMA (2012). Radioactive holmium acetylacetonate microspheres for interstitial microbrachytherapy: an in vitro and in vivo stability study. Pharm Res.

[32] Bult W, Kroeze SGC, Elschot M, Seevinck PR, Beekman FJ, de Jong HWAM (2013). Intratumoral administration of holmium-166 acetylacetonate microspheres: antitumor efficacy and feasibility of multimodality imaging in renal cancer. PLoS One.

[33] Li CC, Chi JL, Ma Y, Li JH, Xia C-Q, Li L, et al. (2014) Interventional therapy for human breast cancer in nude mice with 131I gelatin microspheres (131I–GMSs) following intratumoral injection. Radiat Oncol 9(1). C.Q. Xia, College of Chemistry, Sichuan University, Chengdu (610041), China 10.1186/1748-717X-9-144 PMC4083354 24958442

[34] Chi JL, Li CC, Xia CQ, Li L, Ma Y, Li J-H (2014). Effect of 131I gelatin microspheres on hepatocellular carcinoma in nude mice and its distribution after intratumoral injection. Radiat Res.

[35] Luboldt W, Pinkert J, Matzky C, Wunderlich G, Kotzerke J (2009). Radiopharmaceutical tracking of particles injected into tumors: a model to study clearance kinetics. Curr Drug Deliv United Arab Emirates.

[36] Lee I (1999). Enhanced tumor targeting by an intratumoral injection of colloidal chromic 32P in two human tumors (AsPC-1 pancreas and Ls174T colon) in nude mice. J Surg Oncol.

[37] Lee I, Lee YH (1999). The effect of various therapeutic solutions including colloidal chromic 32P via an intratumoral injection on the tumor physiological parameters of AsPC-1 human pancreatic tumor xenografts in nude mice. Clin Cancer Res.

[38] Lee I, Wallner PE (1997). Evaluation of cellular uptake, tumor retention, radiation response, and tumor pathophysiology in experimental solid tumors after an intratumoral infusion of colloidal 32P. Cancer.

[39] Nguyen H, Ghanem G, Morandini R, Verbist A, Larsimont D, Fallais C (1997). Tumor type and vascularity: important variables in infusional brachytherapy with colloidal 32P. Int J Radiat Oncol Elsevier.

[40] Order SE, Siegel JA, Lustig RA, Principato R, Zeiger LS, Johnson E (1994). A new method for delivering radioactive cytotoxic agents in solid cancers. Int J Radiat Oncol Biol Phys.

[41] Junfeng Y, Duanzhi Y, Xiaofeng M, Zili G, Jiong Z, Yongxian W (1999). [188Re]rhenium sulfide suspension: a potential radiopharmaceutical for tumor treatment following intra-tumor injection. Nucl Med Biol.

[42] Junfeng Y, Ruping Z, Xinlan D, Xiaofeng M, Jianying X, Weiqing H (2000). Intratumoral injection with [(188)re]rhenium sulfide suspension for treatment of transplanted human liver carcinoma in nude mice. Nucl Med Biol.

[43] Watanabe N, Oriuchi N, Igarashi H, Higuchi T, Yukihiro M, Fukushima Y (1997). Preparation of yttrium-90-labeled human macroaggregated albumin for regional radiotherapy. Nucl Med Biol.

[44] Nijsen JFW, Krijger GC, van Het Schip AD (2007). The bright future of radionuclides for cancer therapy. Anti Cancer Agents Med Chem.

[45] Meenan J, Mesenas S, Douglas N, Heatley S, Doig L, Ross P (2007). EUS-delivered therapy for pancreatic cancer: initial experience with targeted injection of 32P Biosilicon™. Gastrointest Endosc Elsevier.

[46] Flocks RH, Culp D, Elkins HB (1956). Treatment of cancer of prostate by interstitial injection of Au 198: studies in problem of distribution. Trans Am Assoc Genitourin Surg.

[47] Lee JD, Yang WI, Lee MG, Ryu YH, Park JH, Shin KH (2002). Effective local control of malignant melanoma by intratumoural injection of a beta-emitting radionuclide. Eur J Nucl Med Mol Imaging.

[48] Sjoholm H, Ljunggren K, Adeli R, Brun A, Ceberg C, Strand SE (1995). Necrosis of malignant gliomas after intratumoral injection of 201Tl in vivo in the rat. Anti-Cancer Drugs.

[49] Kim JK, Han KH, Lee JT, Paik YH, Ahn SH, Lee JD (2006). Long-term clinical outcome of phase IIb clinical trial of percutaneous injection with holmium-166/chitosan complex (Milican) for the treatment of small hepatocellular carcinoma. Clin Cancer Res Clinical Cancer Research.

[50] Ciarrocchi E, Belcari N (2017). Cerenkov luminescence imaging: physics principles and potential applications in biomedical sciences. EJNMMI Physics.

[51] Ma X, Wang J, Cheng Z (2014). Cerenkov radiation: a multi-functional approach for biological sciences. Front Phys.

[52] Carpenter CM, Ma X, Liu H, Sun C, Pratx G, Wang J (2014). Cerenkov luminescence endoscopy: improved molecular sensitivity with β-emitting radiotracers. J Nucl Med.

[53] Chakraborty S, Sharma KS, Rajeswari A, Vimalnath KV, Sarma HD, Pandey U (2015). Radiolanthanide-loaded agglomerated Fe_3_O_4_ nanoparticles for possible use in the treatment of arthritis: formulation, characterization and evaluation in rats. J Mater Chem B.

